# Cell adhesion molecules have prognostic potential in colorectal carcinoma

**DOI:** 10.1186/1471-2164-15-S2-P13

**Published:** 2014-04-02

**Authors:** Asma’a AL-Attas, Abdulrhaman AlMalki, Mamdooh Gari, Shilu Mathew, Jaudah Al-Maghrabi, Mahmoud Al-Ahwal, Mohammed Al-Qahtani, Ashraf Dallol, Abdelbaset Buhmeida

**Affiliations:** 1 Center of Excellence in Genomic Medicine Research, King Abdulaziz University, Jeddah, Kingdom of Saudi Arabia; 2 Faculty of Science, Department of Biochemistry, King Abdulaziz University, Jeddah, Kingdom of Saudi Arabia; 3 Department of Oncology and Scientific Chair of CRC, King AbdulAziz University, Jeddah, Saud Arabia

## Background

The cadherin–catenin complex is vital cell adhesion molecules and it plays a fundamental role in maintaining the cells differentiation and the normal architecture of epithelial tissues [1]. Consequently, the disruption of this complex has been shown in carcinomas and correlated with various pathologic and clinical features. This study seeks for molecular markers in colorectal cancer (CRC) that defines those patients at high risk of recurrence and poor outcome. This study is aimed to assess the expression of selected group of cadherin-catenin; E-cadherin, N-cadherin, α-catenin and β-catenin in subset of primary CRC and determine their relation to different clinico-pathological factors and survival [2].

## Materials and methods

To achieve this, paraffin blocks of 103 CRC patients were retrieved. The expression of E-cadherin, N-cadherin, α-catenin and β-catenin were analyzed by immunohistochemmistry (IHC) and promoter hypermethylation of E-cadherin gene CDH1 was analyzed using Methy-Light Assay. Statistical analyses were performed to determine the association of these markers with clinico-pathological variables.

## Results

The results showed that the expressions of both α-catenin and N-cadherin were significant sign of poor outcome and recurrence as evaluated by univariate Kaplen-Meier for disease-free survival (DFS) (P=0.034, P=0.053). In multivariate survival (Cox) analysis, α-catenin was significantly independent predictor of DFS (P=0.008). On the other hand, β- catenin and E-cadherin exhibits no prognostic power for recurrence and poor survival. Promoter methylation of CDH1 was observed in 45% of our samples and correlation with E-cadherin expression revealed a 60% concordance.

## Conclusions

These results implicate the usefulness of α-catenin and N-cadherin in predicting outcome of patients with CRC.

*This work was financially supported by King Abdulaziz City for Science and Technology* (*KACST*) *under research no* (*ARP -30-262*)*.*
